# New dolutegravir resistance pattern identified in a patient failing antiretroviral therapy

**DOI:** 10.7448/IAS.17.4.19749

**Published:** 2014-11-02

**Authors:** Andreas Carganico, Stefan Dupke, Robert Ehret, Thomas Berg, Axel Baumgarten, Martin Obermeier, Hauke Walter

**Affiliations:** 1 Praxis, MIB – Medical Infectiology Center Berlin, Berlin, Germany; 2 Laboratory Berg, MIB – Medical Infectiology Center Berlin, Berlin, Germany

## Abstract

**Introduction:**

The most recently approved antiretroviral, the integrase inhibitor dolutegravir (DTG), is described to be a very potent drug with a unique resistance profile, but a certain degree of cross-resistance to RAL or EVG induced drug resistance, which is mediated mainly by integrase mutations at positions 140 and 148. The impact of a single N155H mutation to DTG resistance is described to be minor. However, there is only rare data available about the impact of N155H in the context of secondary site integrase mutations. Here, we present a case of virological failure in a DTG treated patient based on N155H mutation background.

**Methods:**

Therapy monitoring of an HIV-HCV co-infected patient harbouring already an omni-drug-class resistant HIV-1 in consequence of more than 20 years ART history. Drug susceptibility testing was performed by RT-PCR from plasma and subsequent Sanger sequencing. Tropism testing was done from proviral DNA with FPR cut-offs according to the German recommendations.

**Results:**

In 2013, the patient harbouring a virus with high level resistance to all RTI and PI received a regimen containing FTC, TDF, DRV/r, RPV, T20, and RAL to handle a viral load of 5000 RNA copies/mL, but never achieved fully suppressed viral load. In June 2013, after S119R, N155H and E157Q mutations in viral integrase were detected, the patient received DTG, and RAL was stopped. One month later, when viral load was undetectable for the first time since 2007, ART was de-escalated by removing T20. Since February 2014, low-level viral load was re-detectable. Two new mutations T97A and S147G in the integrase and no other new resistance associated mutations in protease and reverse transcriptase in comparison to the sample analyzed in June 2013 were detected.

**Conclusions:**

Highly resistant HIV-1 strains have been a common problem in the past. Their frequencies were pushed back by highly potent ART, but the virus is still able to become resistant against all available antiretrovirals at once. The here documented strain became resistant to DTG without carrying mutations at the described positions 140 and 148, but in the context of integrase N155H. Since N155H alone is described not to contribute sufficient resistance to DTG, there seems to be a need to re-check viruses with N155H plus minor mutations (like T97A, S119R, S147G and E157Q) potentially contributing to DTG resistance.

